# Steroid receptor coactivator 1 deficiency increases MMTV-neu mediated tumor latency and differentiation specific gene expression, decreases metastasis, and inhibits response to PPAR ligands

**DOI:** 10.1186/1471-2407-10-629

**Published:** 2010-11-16

**Authors:** Ji Seung Han, David L Crowe

**Affiliations:** 1 University of Illinois Cancer Center, 801 S. Paulina Street, Room 530C, Chicago, IL 60612, USA

## Abstract

**Background:**

The peroxisome proliferator activated receptor (PPAR) subgroup of the nuclear hormone receptor superfamily is activated by a variety of natural and synthetic ligands. PPARs can heterodimerize with retinoid X receptors, which have homology to other members of the nuclear receptor superfamily. Ligand binding to PPAR/RXRs results in recruitment of transcriptional coactivator proteins such as steroid receptor coactivator 1 (SRC-1) and CREB binding protein (CBP). Both SRC-1 and CBP are histone acetyltransferases, which by modifying nucleosomal histones, produce more open chromatin structure and increase transcriptional activity. Nuclear hormone receptors can recruit limiting amounts of coactivators from other transcription factor binding sites such as AP-1, thereby inhibiting the activity of AP-1 target genes. PPAR and RXR ligands have been used in experimental breast cancer therapy. The role of coactivator expression in mammary tumorigenesis and response to drug therapy has been the subject of recent studies.

**Methods:**

We examined the effects of loss of SRC-1 on MMTV-neu mediated mammary tumorigenesis.

**Results:**

SRC-1 null mutation in mammary tumor prone mice increased the tumor latency period, reduced tumor proliferation index and metastasis, inhibited response to PPAR and RXR ligands, and induced genes involved in mammary gland differentiation. We also examined human breast cancer cell lines overexpressing SRC-1 or CBP. Coactivator overexpression increased cellular proliferation with resistance to PPAR and RXR ligands and remodeled chromatin of the proximal epidermal growth factor receptor promoter.

**Conclusions:**

These results indicate that histone acetyltransferases play key roles in mammary tumorigenesis and response to anti-proliferative therapies.

## Background

The peroxisome proliferator activated receptor (PPAR) subgroup of the nuclear hormone receptor superfamily are activated by a variety of ligands such as fatty acids, prostaglandin J_2 _metabolites, fibrates, and thiazolidinedione drugs [for review see [1,2]]. Clofibrate is approved for treatment of hyperlipidemia while ciglitazone analogs are used as antidiabetic drugs. Both classes of drugs have been used as experimental cancer therapies. PPARs have functional domains for DNA and ligand binding and interact with recognition sequences in the promoter regions of their target genes to regulate transcription [3]. PPARs can heterodimerize with retinoid X receptors, which have homology to other members of the nuclear receptor superfamily [4]. Natural and synthetic ligands for RXRs, such as, AGN194204 have been characterized, and heterodimerization with PPARs greatly enhances DNA binding and transcriptional activation [5-7]. RXR selective ligands were highly effective in preclinical models of mammary cancer.

Ligand binding to PPARs results in recruitment of transcriptional coactivator proteins such as steroid receptor coactivator 1 (SRC-1) and CREB binding protein (CBP). Both SRC-1 and CBP are histone acetyltransferases, which by modifying nucleosomal histones, produce more open chromatin structure and increase transcriptional activity [8,9]. Members of the SRC family (SRC-1, -2, and -3) share ~40% sequence identity, a basic helix-loop-helix domain involved in dimerization and DNA interaction, and a PAS domain for protein interaction. LXXLL motifs, which allow binding to nuclear receptors, are located in the central region of these proteins [10]. Nuclear hormone receptors can recruit limiting amounts of coactivators from other transcription factor binding sites such as AP-1, thereby inhibiting the activity of AP-1 target genes [11]. Expression of the SRC family member SRC-3 is amplified or overexpressed in breast and ovarian cancer [12,13]. Translocation of the SRC-2 and monocytic zinc finger (MOZ) gene has been demonstrated in acute myeloid leukemia [14]. SRC-1 null mutant mice are viable and fertile but exhibit partial resistance to many hormones including estrogen, progestin, androgen, and thyroid [15,16]. We previously demonstrated that SRC-1 expression imparted estrogen responsiveness to non-reproductive tract cancer cell lines [17].

PPAR and RXR ligands have been used in experimental cancer therapies [18]. Human breast cancer cells express PPARs and RXRs, and ligands for these receptors have been shown to induce growth inhibition and apoptosis in cell cultures and animal models [19-24]. These PPAR and RXR ligands generally did not produce dose limiting side effects, but clinical trials failed to show objective responses in patients with advanced breast cancer [25,26]. The role of coactivator expression in this lack of response to drug therapy has recently received attention. A previous study from our laboratory demonstrated that coactivator overexpression produced resistance to the nuclear hormone retinoic acid [27]. Similarly altered SRC-1 expression has been associated with resistance to anti-estrogen therapy and CBP is overexpressed in some breast cancers [28,29]. Previous studies demonstrated decreased metastasis in tumors driven by polyoma middle T antigen in SRC1-/- mice [30]. To understand how coactivator proteins regulate response to nuclear hormone receptor ligands such as PPAR and RXR we examined neu oncogene driven mammary tumorigenesis in mice lacking SRC-1. We also used a targeted gene approach in human breast cancer cell lines overexpressing SRC-1. SRC-1 null mutation in mice overexpressing the neu oncogene in mammary epithelium significantly increased the tumor latency period, reduced tumor proliferation index and metastasis, inhibited response to PPAR and RXR ligands, and induced differentiation specific gene expression. Coactivator overexpression in human breast cancer cell lines increased proliferation with resistance to PPAR and RXR ligands and remodeled chromatin of the proximal epidermal growth factor receptor promoter, which is a principal growth signaling pathway in mammary epithelial cells. These results indicate that SRC-1 expression plays key roles in mammary tumorigenesis and response to anti-proliferative therapies.

## Methods

### Transgenic Mouse Procedures

Animal procedures were approved by the institutional animal care committee. SRC1-/- mice (15) were crossed with the mammary tumor prone MMTV-neu transgenic strain in the FVB background (The Jackson Laboratory). These offspring were bred back to the SRC1-/- strain to create SRC1-/- mice harboring the neu proto-oncogene. MMTV-neu mice were also bred with SRC1+/+ control mice. Mice were genotyped by PCR analysis of tail DNA samples. Mice were given P.O. doses of 100 mg/kg clofibrate, ciglitazone, AGN194204, or vehicle in corn oil daily for 8 weeks (20 mice/group). The mammary gland chains of female mice were examined visually and by palpation twice weekly. Tumors were measured twice weekly using calipers. Mice were euthanized when tumors reached 2 cm in largest dimension. Tumor tissue was processed for histopathologic and gene expression analyses. Statistical analysis was determined by ANOVA.

### Histopathology and Immunohistochemistry

Tumor tissue was fixed in formalin for 16 hours at room temperature. Tissue was dehydrated in an ethanol series followed by clearing in xylene and embedding in paraffin. Seven micrometer sections were cut from the blocks and placed on poly-L-lysine coated slides. Sections were deparaffinized in xylene and stained with hematoxylin and eosin for histopathologic interpretation. For immunohistochemistry, sections were rehydrated and blocked with 10% normal serum followed by incubation with anti-PCNA antibody for one hour at room temperature. After washing in PBS, the sections were incubated with anti-mouse IgG secondary antibody conjugated to biotin for 10 minutes at room temperature. After additional washing in PBS, the cultures were incubated with streptavidin conjugated horseradish peroxidase enzyme for 10 minutes at room temperature. Following final washes in PBS, antigen-antibody complexes were detected by incubation with hydrogen peroxide substrate solution containing aminoethylcarbazole chromogen reagent. Immunohistochemical results were photographed using light microscopy. The percentage of PCNA positive cells were determined by counting cells in 10 random high power fields. Statistical significance was determined by t test.

### Gene Expression Profiling

Total RNA was extracted from SRC1+/+ and SRC1-/- tumors using a commercially available kit (RNEasy, Qiagen, Valencia, CA). Integrity of ribosomal RNA bands was confirmed by northern gel electrophoresis. Total RNA (10 μg) was converted to labeled cRNA targets. The biotinylated cRNA targets were then purified, fragmented, and hybridized to GeneChip mouse genome 430 2.0 expression arrays (Affymetrix, Santa Clara, CA) to interrogate the abundance of 39,000 possible transcripts in each sample. Affymetrix GCOS software was used to generate raw gene expression scores and normalized to the relative hybridization signal from each experiment. All gene expression scores were set to a minimum value of 2 times the background determined by GCOS software in order to minimize noise associated with less robust measurements of rare transcripts. Normalized gene expression data was imported into dChip software http://www.biostat.harvard.edu/complab/dchip for hierarchical clustering analysis using the average linkage algorithm. Data was analyzed by t test with p < 0.005 followed by ratio analysis (minimum 2 fold change).

### Cell Culture and Stable Transfection

The human breast cancer cell lines used in this study were purchased from the American Type Culture Collection and cultured in Dulbecco's modified Eagle medium, 10% fetal bovine serum, and 40 μg/ml gentamicin in a humidified atmosphere of 5% CO_2 _at 37°C. Cultures were treated with 100 μM clofibrate (PPARα selective agonist), ciglitazone (PPARγ selective agonist), or 100 nM AGN194204 (RXR selective agonist; kindly provided by Dr. Roshantha Chandraratna) or vehicle for up to 24 hours. MCF7 and MDA-MB-468 cells were transfected with 2 μg SRC-1, CBP, or neomycin resistance plasmid using Lipofectamine according to manufacturer's recommendations (Invitrogen). Cells were selected in 400 μg/ml G418 for 14 days. Resistant clones were picked for expansion and characterization.

### Cell Proliferation Analysis

Triplicate cultures of 3 × 10^4 ^parental, SRC-1, CBP, or vector control clones were plated into six well plates and treated with 100 μM clofibrate or ciglitazone, 100 nM AGN194204, or vehicle alone or in combination for 6 days. Cultures were trypsinized and counted at 2 day intervals using a hemacytometer.

### Western Blot

Protein was extracted in 1× Laemmli buffer from human breast cancer cell lines and clones treated with PPAR or RXR ligands. Seventy-five μg total cellular protein was separated by SDS-PAGE on 10% resolving gels under denaturing and reducing conditions. Separated proteins were electroblotted to PVDF membranes according to manufacturer's recommendations (Roche Applied Science). Blots were incubated with antibodies to PPARs, RXRα, SRC-1, CBP, EGFR, ERK1, c-myc, cyclin A, cyclin E, cdk1, cdk4, casein, WAP, or β-actin for 16 hours at 4°C. After washing in Tris buffered saline containing 0.1% Tween 20 (TBST, pH 7.4), blots were incubated for 30 minutes at room temperature with anti-IgG secondary antibody conjugated to horseradish peroxidase. Following extensive washing in TBST, bands were visualized by the enhanced chemiluminescence method (Roche Applied Science). Bands were quantitated by laser densitometry.

### Chromatin Immunoprecipitation

Parental and neomycin resistant MCF7 and MDA-MB-468 human breast cancer cells and those overexpressing SRC-1 or CBP proteins were treated with 100 μM clofibrate or ciglitazone, 100 nM AGN194204, or vehicle for 24 hours. After washing in PBS, cells were fixed in 1% formaldehyde for 10 minutes at room temperature. Cells were washed in PBS and lysed in immunoprecipitation buffer containing protease inhibitors for 30 minutes at 4°C, sheared, and centrifuged at 10,000 × g for 10 minutes. Supernatants were cleared with 2 μg sheared salmon sperm DNA, 20 μl preimmune serum, and 20 μl protein A/G sepharose beads for 2 hours at 4°C. Aliquots of the supernatant were used as input DNA for normalization. Immunoprecipitation using anti-SRC-1, anti-CBP, or anti-acetylated histone H3 antibodies (Santa Cruz Biotechnology) was performed overnight at 4°C. Preimmune IgG was used as the negative control antibody. Immunoprecipitates were washed extensively in immunoprecipitation buffer, resuspended in 10 mM Tris-HCl, 1 mM EDTA (TE, pH 8) and incubated at 65°C for 6 hours to reverse crosslinks. The supernatants were extracted with phenol/chloroform and ethanol precipitated. Following washing in 70% ethanol, pellets were dried and suspended in 50 μl TE. For real time PCR, 1 μl of template was amplified in buffer containing 10 mM Tris-HCl (pH 8.3), 50 mM KCl, 2.5 mM MgCl_2_, 200 nM each dNTP, and 100 ng each primer (5'-GCCTCCGCCCCCCGCACGGTG-3' and 5'-CGCTGCTCCCCGAAGAGCTCG-3') flanking the proximal EGFR promoter (-221 bp to -1 bp; 30). The optimized cycle parameters were one cycle at 94°C for 3 minutes followed by 25 cycles of 94°C for 25 seconds, 58°C for 60 seconds, 72°C for 60 seconds, and one final cycle at 72° for 10 minutes (iCycler, Bio-Rad).

### Nucleosomal Mapping

Nuclei were isolated from parental human breast cancer cell lines MCF7 and MDA-MB-468 and coactivator overexpressing clones treated with 100 μM clofibrate or ciglitazone, 100 nM AGN194204, or vehicle for 24 hours. Chromatin was digested to mononucleosomal form with micrococcal nuclease (Roche Applied Science). The digestion was stopped by addition of 50 mM EDTA. Nuclei were lysed in 1% SDS and treated with 0.1 mg proteinase K overnight at 37°C. DNA was purified by phenol/chloroform extraction and ethanol precipitation. DNA was suspended in TE buffer and analyzed by agarose gel electrophoresis to ensure digestion to mononucleosomal fragments. These fragments are eluted from the gel and used as PCR templates to determine nucleosomal occupancy of the proximal EGFR promoter as described in the preceding section. Undigested genomic DNA was used as the positive control and template free samples were used as the negative control.

### Transient Transfection and Promoter Activation

Triplicate cultures of 50% confluent MCF-7 or MDA-MB-468 cells were transiently transfected with 5 μg of the human EGFR promoter/reporter vectors along with 2 μg SRC-1, CBP, or blank expression plasmids using Lipofectamine according to the manufacturer's recommendations (Invitrogen). One μg β-galactosidase expression plasmid was used to normalize for transfection efficiency. Cultures were treated with 100 μM clofibrate or ciglitazone, 100 nM AGN194204, or vehicle for 24 hours. Cells were harvested and reporter gene activity determined using a commercially available kit and luminometer (Applied Biosystems). Luciferase activity was normalized to β-galactosidase levels for each sample. Statistical significance was determined by ANOVA.

### Reverse Transcription-Polymerase Chain Reaction

RNA was extracted from SRC-1 and CBP clones using a commercially available kit (Qiagen) and reverse transcribed using SuperScript II reverse transcriptase according to manufacturer's instructions (Invitrogen). cDNA was amplified using specific primers (PPARα 5'-TGACCTGGCCCTATTCATTG-3' and 5'-GTAGATCTCCTGGAGCAGAG-3'; PPARγ 5'-TTTGCTGAATGTGAAGCCC-3' and 5'-GTGAAGACTCATGTCTGTCTC-3'; RXRα 5'-TCTTTAACCCTGACTCCAAGG-3' and 5'-GCCTCCAGCATCTCCATAAG-3'; CBP 5'-CACACCCACACACATCTATC-3' and 5'-ACAAAAAACCCCGAACACTAAG-3'; SRC-1 5'-ACTGCAACCAGCTCTCATCC-3' and 5'-TTGCTCCAAATGCTACGACC-3') in 20 mM Tris-HCl (pH 8.3), 1.5 mM MgCl_2_, 63 mM KCl, 0.05% Tween 20, 1 mM EGTA, 50 μM of each dNTP, and 2.5 U Taq DNA polymerase (Roche Applied Science). Amplification with β-actin cDNA using primers 5'-ACAGGAAGTCCCTTGCCATC-3' and 5'-ACTGGTCTCAAGTCAGTGTACAGG-3' as the internal control was carried out by real time PCR (iCycler, Bio-Rad) using cycle parameters 94°C for 25 seconds, 55°C for 1 minute, and 72°C for 1 minute.

## Results

**Figure 1:**
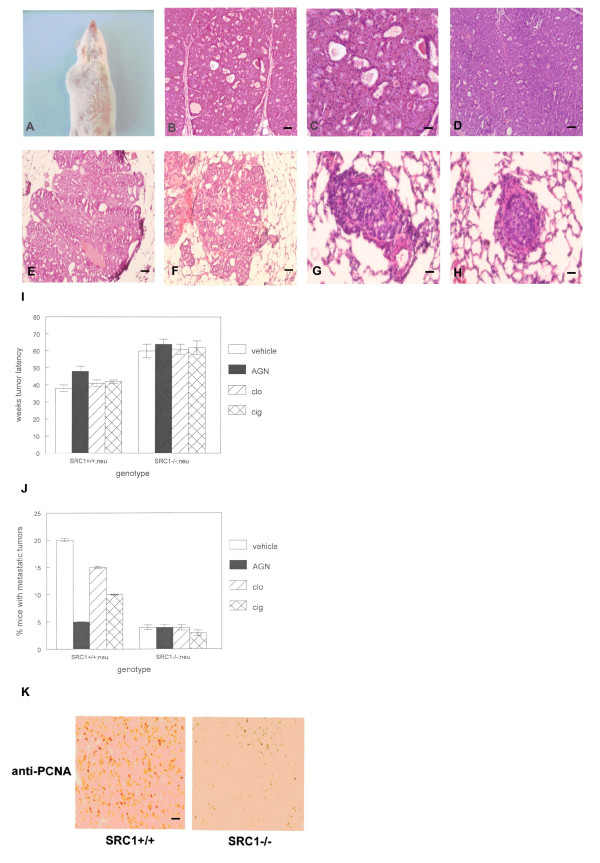
**Increased tumor latency and decreased metastasis in oncogene induced mammary tumors from SRC-1 null mutant mice**. (A) Mammary tumor formation (at left) in the MMTV-neu mouse. (B, C) The histopathologic appearance of mammary tumors from the SRC1-/-;neu mouse resembles poorly differentiated adenocarcinoma in humans. Hematoxylin and eosin stained sections are shown. Scale bars 100 μm and 50 μm respectively. (D) Histopathologic appearance of human poorly differentiated adenocarcinoma stained with hematoxylin and eosin. Scale bar = 50 μm. (E, F) Histopathologic appearance of early mammary tumors (prior to detection by palpation) in SRC1+/+;neu and SRC1-/-;neu mice is consistent with poorly differentiated adenocarcinoma. Scale bar = 100 μm. (G, H) Histopathologic appearance of SRC1+/+;neu and SRC1-/-;neu mammary tumor lung metastases is consistent with poorly differentiated adenocarcinoma. Scale bars = 50 μm. (I) Increased tumor latency in SRC1-/-;neu mice compared to SRC1+/+;neu animals. Mice of both genotypes were treated with AGN194204 (AGN), clofibrate (clo), or ciglitazone (cig) as described in Materials and Methods. The mammary gland chains of mice were examined weekly by palpation to detect tumors. (J) Decreased mammary tumor metastasis in SRC1-/- mice. SRC1+/+;neu and SRC1-/-;neu mice were treated with 100 mg/kg AGN194204, clofibrate, or ciglitazone as described in Materials and Methods. The number of metastatic lung lesions was counted at necropsy in each group of mice. (K) Loss of SRC-1 expression inhibits proliferation index in oncogene induced mammary tumors. Tumor sections from SRC1+/+;neu and SRC1-/-;neu were subjected to immunohistochemistry using anti-PCNA antibody. These experiments were performed 3 times with similar results. Representative sections are shown.

SRC-1 knockout animals are viable and fertile [15]. To examine the effects of SRC-1 null mutation on mammary tumorigenesis in vivo, we created SRC1-/-;neu and SRC1+/+;neu mice and characterized mammary tumors arising in these animals following administration of PPAR or RXR ligands. The gross appearance of mammary tumors in vivo is shown in Figure 1A. Neither the SRC-1 null mutation nor ligand treatment affected the histopathologic pattern of these tumors (Figure 1B, C). Tumors showed abnormal ducts containing secretory products separated by sheets of poorly differentiated epithelial cells. This pattern was similar to poorly differentiated adenocarcinoma in human breast cancer (Figure 1D) as previously reported for MMTV-neu mice [31]. Very early stage tumors (prior to detection by palpation) also demonstrated a histopathologic pattern consistent with poorly differentiated adenocarcinoma (Figure 1E, F). The histopathologic appearance of metastatic tumors in the lungs of SRC1+/+;neu and SRC1-/-;neu mice was consistent with poorly differentiated adenocarcinoma (Figure 1G, H). The SRC-1 null mutation dramatically increased the mean tumor latency period in these mice compared to controls (60 vs. 38 weeks; p < 10^-10^; Figure 1I). Treatment with AGN194204 extended the tumor latency period to 48 weeks in SRC1+/+ mice (p < 0.001). Clofibrate and ciglitazone treatment extended the latency period to 41 and 42 weeks respectively in SRC1+/+ mice (p < 0.05 for ciglitazone). The increased tumor latency period due to ligand treatment was not observed in SRC1-/- mice, indicating that SRC-1 was important for these effects. SRC1-/-mice had significantly fewer metastatic tumors than SRC1+/+ animals (80% reduction; p < 0.002; Figure 1J). Treatment with AGN194204, clofibrate, and ciglitazone reduced the number of metastatic tumors in SRC1+/+ mice (20-80% reduction; p < 0.02). No statistically significant differences in growth rates or number of tumors were observed between the groups. These results indicate that SRC-1 null mutation increased mammary tumor latency, decreased metastasis, and reduced the inhibitory effects of PPAR and RXR ligands in neu transgenic mice.

Despite the distinct biological differences between SRC1-/-;neu and SRC1+/+;neu mammary tumors, there were no apparent histopathologic changes reflective of the change in phenotype. This finding is similar to human breast cancer in which tumors with similar histopathologic diagnoses often manifest different clinical behaviors [32]. This issue has subsequently been addressed by classifying tumors according to their global gene expression profiles. To begin to understand the dramatic effects of the SRC-1 null mutation on inhibition of mammary tumorigenesis in the neu transgenic mouse, we performed global gene expression profiling of tumors from SRC1+/+ and SRC1-/- animals. We identified 237 differentially expressed genes meeting the criteria of p < 0.005 and minimum 2 fold change in expression. As shown in Table 1, SRC1-/- tumors expressed higher mRNA levels of the cyclin dependent kinase inhibitors p21 and p15 (8.8 fold and 8.5 fold increases respectively). Inhibitors of epithelial cell proliferation such as transforming growth factor beta 2 were upregulated in SRC1-/- tumors (5.2 fold). These changes in expression of inhibitors of cell cycle progression may help explain the increased latency period in SRC1-/- tumors. To confirm these results, we examined the proliferation index in SRC1-/- and SRC1+/+ tumors by anti-PCNA immunohistochemistry. As shown in Figure 1K, SRC1-/- tumors showed significantly decreased numbers of PCNA positive cells compared to SRC1+/+ cancers (6% vs. 70%; p < 0.0002). SRC1-/- tumors also highly expressed differentiation markers (parotid secretory protein, 313.4 fold; salivary amylase 1, 242.1 fold; prolactin induced protein, 71.9 fold; casein alpha s2-like A, 13.8 fold; whey acidic protein, 7.1 fold; demilune cell and parotid protein, 6.7 fold). These data indicate that SRC1-/- mammary tumors are less proliferative and more differentiated with respect to gene expression than SRC1+/+ cancers.

**Table 1:** Differentially expressed genes between SRC1+/+ and SRC1-/- mammary tumors (237 genes)

**Accession**	**Gene symbol**	**Gene name**	**fold change**
BC010288	Psp	parotid secretory protein	313.4
NM_007446	Amy1	amylase 1, salivary	242.1
BC011134	Chia	chitinase, acidic	226.6
NM_011422	Smr1	submaxillary gland androgen regulated protein	209.7
NM_008843	Pip	prolactin induced protein	71.9
AF108501	Clca1	chloride channel calcium activated 1	42.7
BG862223	Camk2b	Calcium/calmodulin-dependent protein kinase II, b	39.5
NM_009323	Tbx15	T-box 15	38.3
NM_009638	Crisp1	cysteine-rich secretory protein 1	35.4
BB795585	Ntrk2	neurotrophic tyrosine kinase, receptor, type 2	21.7
NM_013605	Muc1	mucin 1, transmembrane	17.2
BF119305	Csn1s2a	casein alpha s2-like A	13.8
NM_008109	Gdf5	growth differentiation factor 5	10.9
NM_053134	Pcdhb9	protocadherin beta 9	9.7
AK007630	Cdkn1a	cyclin-dependent kinase inhibitor 1A (p21)	8.8
AF059567	Cdkn2b	cyclin-dependent kinase inhibitor 2B (p15)	8.5
BF683028	Gyk	glycerol kinase	7.2
NM_011709	Wap	whey acidic protein	7.1
C86550	Dcpp	demilune cell and parotid protein	6.7
NM_007554	Bmp4	bone morphogenetic protein 4	6.1
BF456404	Pak1	p21 (CDKN1A)-activated kinase 1	5.4
AV246759	Tgfb2	transforming growth factor, beta 2	5.2
NM_007865	Dll1	delta-like 1 (Drosophila)	5.0
NM_010669	Krt6b	Keratin 6B	5.0
BQ032637	Jak1	Janus kinase 1	-3.5
BG064038	Arhgap12	Rho GTPase activating protein 12	-3.5
BC021401	Muc10	mucin 10, submandibular gland salivary mucin	-3.9
AK020693	Acpp	acid phosphatase, prostate	-4.1
BB524685	Tmprss11a	transmembrane protease, serine 11a	-5.6
NM_009973	Csn1s2b	casein alpha s2-like B	-5.9
U82380	Smr2	submaxillary gland androgen regulated protein 2	-6.0
NM_013655	Cxcl12	chemokine (C-X-C motif) ligand 12	-6.4
BF011461	Rbbp4	retinoblastoma binding protein 4	-6.4
BB745167	Ppargc1a	PPAR, gamma, coactivator 1 alpha	-7.2
NM_011271	RNase 1	ribonuclease, RNase A family, 1 (pancreatic)	-9.4
BB760085	Eya1	eyes absent 1 homolog (Drosophila)	-9.4
AV334599	Tcfap2b	transcription factor AP-2 beta	-33.6
AV275795	Plb1	phospholipase B1	-47.4
BC021770	Cldn10	claudin 10	-81.2

**Figure 2:**
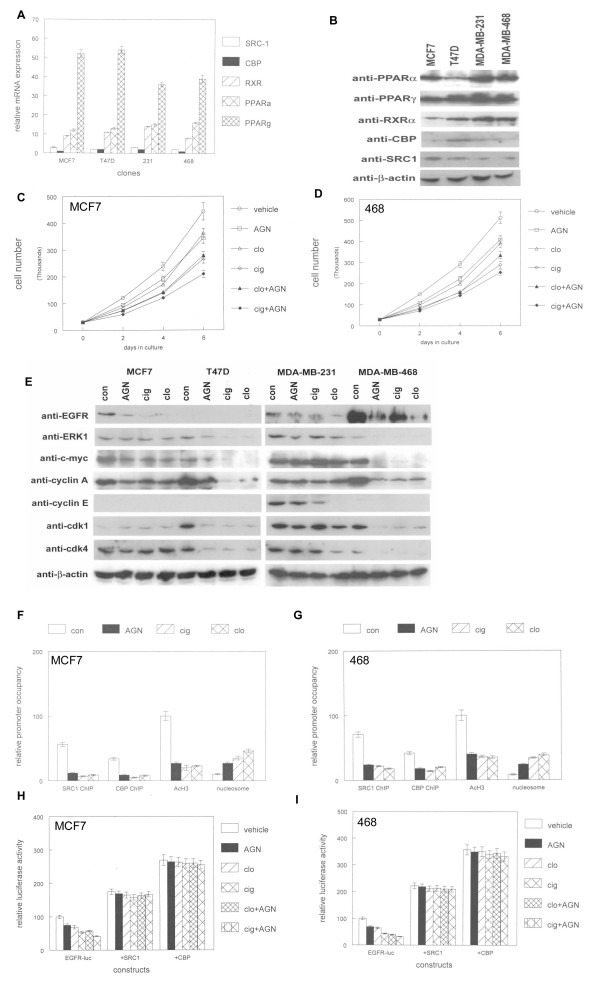
**PPAR and RXR ligands inhibit human breast cancer cell proliferation in vitro**. (A) Expression of SRC-1, CBP, RXRα, and PPAR mRNA in human breast cancer cell lines. (B) Expression of SRC-1, CBP, RXRα, and PPAR proteins in human breast cancer cell lines. (C, D). Triplicate cultures of MCF7 or MDA-MB-468 cells were treated with 100 μM of the PPARα agonist clofibrate (clo) or the PPARγ agonist ciglitazone (cig) alone or in combination with 100 nM of the RXR selective compound AGN194204 or vehicle for 6 days. At 2 day intervals, cells were trypsinized and counted using a hemacytometer. Error bars represent SEM of 3 independent experiments. (E) Human breast cancer cell lines MCF7, T47 D, MDA-MB-231, and MDA-MB-468 were treated with 100 nM AGN194204, 100 μM ciglitazone (cig) or clofibrate (clo), or vehicle for 16 hours. Protein extracts from these treated cells were subjected to western blotting using cell cycle antibodies indicated at left. These experiments were performed 3 times with similar results. (F, G) PPAR and RXR ligands inhibit coactivator occupancy of the proximal EGFR promoter. MCF7 or MDA-MB-468 cells were treated with 100 nM AGN194204, 100 μM ciglitazone (cig) or clofibrate (clo), or vehicle for 16 hours followed by chromatin immunoprecipitation (ChIP) with antibodies to SRC1, CBP, or acetylated histone H3 (acH3). Nuclease digested DNA from treated cells was amplified to detect nucleosomal loading of the EGFR promoter. The proximal promoter region was amplified by quantitative PCR and amplification products normalized to input DNA. (H, I) SRC-1 overexpression blocks PPAR and RXR ligand mediated suppression of EGFR promoter activity. The EGFR-luc promoter/reporter vector was transiently transfected with SRC-1, CBP, or control expression vectors into triplicate cultures of MCF7 or MDA-MB-468 cells. Cultures were treated with AGN194204, clofibrate, or ciglitazone alone or combination. Luciferase activity was determined as described in Materials and Methods. Error bars indicate SEM of 3 independent experiments.

We also determined the effects of SRC-1 on PPAR and RXR mediated proliferation of human breast cancer cell lines. We previously demonstrated that human breast cancer cell lines expressed PPARα and PPARγ, and their lipid ligands inhibited proliferation of these cells [22]. We examined expression of PPARs, RXRα, and their coactivators SRC-1 and CBP by quantitative RT-PCR in human breast cancer cell lines. As shown in Figure 2A, PPARγ was expressed at highest levels in human breast cancer cell lines. PPARα was expressed at 4-5 fold lower levels in these lines. RXRα was expressed at levels similar to that of PPARα. SRC-1 was expressed at levels 10 fold lower than PPARγ and CBP mRNA was expressed at lowest levels in these lines. By western blotting, we determined PPAR, RXRα, coactivator, and cell cycle regulatory protein expression in human breast cancer cell lines. As shown in Figure 2B, PPARγ protein was expressed at highest levels in human breast cancer cell lines. PPARα and RXRα were expressed at lower levels, while SRC-1 and CBP expression was barely detectable by western blot. To examine potential synergistic effects of synthetic PPAR and RXR ligands on these lines, we treated human breast cancer cells with the PPARα agonist clofibrate, the PPARγ selective ligand ciglitazone, and the RXR selective agonist AGN194204. These agents inhibited proliferation of all four cell lines tested (MCF7, T47 D, MDA-MB-231, MDA-MB-468). As shown in Figure 2C and 2D, clofibrate and AGN194204 as single agents inhibited MCF7 and MDA-MB-468 cell proliferation by 20%. Ciglitazone was a more effective inhibitor of proliferation as a single agent than the other two compounds (45-50% fewer cells compared to control cultures). The combination of clofibrate and AGN194204 produced an additive effect on inhibition of breast cancer cell proliferation (40% fewer cells compared to control cultures). The combination of ciglitazone and AGN194204 was the most effective inhibitor of proliferation (55-60% fewer cells compared to control cultures). Cytologic features of programmed cell death (cellular condensation and rounding, membrane blebbing) were not observed during treatment with these ligands. These results indicate that PPAR and RXR selective ligands are effective inhibitors of breast cancer cell proliferation in vitro.

To determine which cell cycle regulators were involved in inhibition of breast cancer cell proliferation by PPAR and RXR ligands, we treated 4 lines individually with these ligands and examined protein expression by western blot. As shown in Figure 2E, expression of EGFR, c-myc, and cyclin A was inhibited by PPAR and RXR ligands in MCF7 cells (up to 10 fold, 4 fold, and 5 fold respectively). Expression of other cell cycle regulatory proteins such as ERK1 was not affected in MCF7 cells. In T47 D cells, expression of ERK1, c-myc, cyclin A, cdk1 and cdk4 proteins were inhibited by PPAR and RXR ligands (4 fold, 10 fold, 11 fold, 5 fold, and 4 fold respectively). In MDA-MB-231 cells, expression of EGFR, ERK1, cyclin E, cdk1, and cdk4 proteins were inhibited (4 fold, 2 fold, 15 fold, 2 fold, and 4 fold respectively) by these ligands. In MDA-MB-468 cells, expression of EGFR, ERK1, c-myc, cyclin A, cdk1, and cdk4 proteins were inhibited (20 fold, 3 fold, 14 fold, 9 fold, 6 fold, and 3 fold respectively) by PPAR and RXR selective ligands. These results indicate that PPAR and RXR ligands inhibit multiple overlapping cell cycle regulatory proteins in human breast cancer cell lines.

Given that nuclear receptors such as PPARs and RXRs mediate control of gene expression via recruitment of coactivator proteins from other transcription factor binding sites such as AP-1 [11], we examined interaction of CBP and SRC-1 histone acetyltransferases within the proximal EGFR promoter, which contains three AP-1 sites by chromatin immunoprecipitation [33]. As shown in Figure 2F and 2G, treatment of MCF7 or MDA-MB-468 cells with PPAR or RXR ligands reduced SRC-1 occupancy of the proximal EGFR promoter by 70-80%. These ligands also reduced CBP occupancy of the promoter by 60-80%. Consistent with this loss of interaction with histone acetyltransferases, acetylation of histone H3 in the proximal EGFR promoter was reduced by 65-75% following treatment with PPAR and RXR ligands. Relative nucleosomal occupancy of the proximal EGFR promoter increased 2-4 fold in cells treated with PPAR or RXR ligands. These changes in chromatin were consistent with loss of EGFR expression and inhibition of proliferation in MCF7 and MDA-MB-468 cells treated with these ligands. These results indicate that PPAR and RXR ligands inhibit interaction of coactivator proteins with key growth regulatory genes such as EGFR.

Given that PPAR and RXR ligand-mediated loss of SRC-1 and CBP occupancy in the proximal EGFR promoter correlated with decreased expression of the receptor, we wished to determine if coactivator overexpression could counteract effects of the ligands. As shown in Figure 2H and 2I, treatment of MCF7 or MDA-MB-468 cells with AGN194204 or clofibrate inhibited activity of the transiently transfected EGFR promoter by 25-30%. Ciglitazone treatment or the combination of clofibrate and AGN194204 further inhibited EGFR promoter activity to 40-50% less than control levels. The combination of ciglitazone and AGN194204 inhibited EGFR promoter activity by 60-70%. However when SRC-1 was transfected with the EGFR reporter construct, promoter activity was induced to 180% of control levels. CBP expression induced EGFR promoter activity to 270% of control levels. Coactivator overexpression completely blocked the inhibitory effects of PPAR and RXR ligands. These results indicate that coactivators can induce resistance to inhibition of EGFR promoter activity by nuclear receptor ligands.

**Figure 3:**
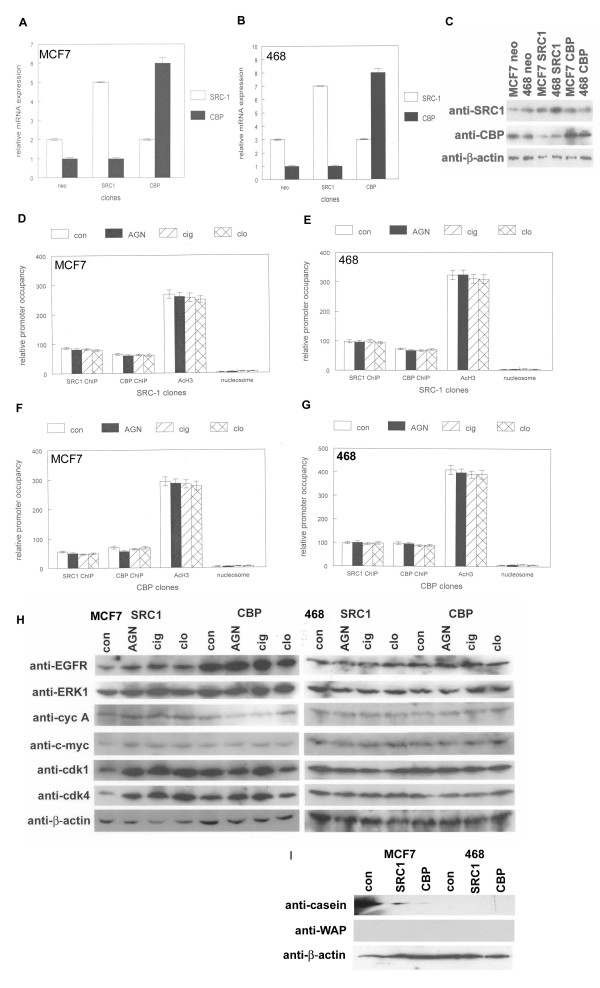
**Coactivator overexpression induces resistance to the anti-proliferative effects of PPAR and RXR ligands**. (A, B) Expression of SRC-1 and CBP mRNAs in stable MCF7 and MDA-MB-468 clones is shown by quantitative RT-PCR. Coactivator expression is normalized to levels of β-actin mRNA. Error bars indicate SEM of 3 independent experiments. (C) Expression of SRC-1 and CBP proteins in human breast cancer clones. (D, E) MCF7 or MDA-MB-468 cells overexpressing SRC- 1 were treated with 100 nM AGN194204, 100 μM ciglitazone (cig) or clofibrate (clo), or vehicle for 16 hours followed by chromatin immunoprecipitation (ChIP) with antibodies to SRC1, CBP, or acetylated histone H3 (acH3). Nuclease digested DNA from treated cells was amplified to detect nucleosomal loading of the EGFR promoter. The proximal promoter region was amplified by quantitative PCR and amplification products normalized to input DNA. (F, G) MCF7 or MDA-MB-468 cells overexpressing CBP were treated with 100 nM AGN194204, 100 μM ciglitazone (cig) or clofibrate (clo), or vehicle for 16 hours followed by chromatin immunoprecipitation (ChIP) with antibodies to SRC1, CBP, or acetylated histone H3 (acH3). Nuclease digested DNA from treated cells was amplified to detect nucleosomal loading of the EGFR promoter. The promoter region was amplified by quantitative PCR. (H) MCF7 (left panel) and MDA-MB-468 (right panel) cells stably expressing SRC-1 or CBP were treated with 100 μM clofibrate (clo) or ciglitazone (cig), 100 nM AGN194204, or vehicle for 16 hours. Protein extracts from these treated cells were subjected to western blotting using cell cycle antibodies indicated at left. These experiments were performed 3 times with similar results. (I) SRC-1 and CBP overexpression inhibits casein protein levels in human breast cancer cells. Protein extracts from MCF7 and MDA-MB-468 clones expressing SRC-1 or CBP or control vector were subjected to western blotting using antibodies indicated at left.

To examine the effects of SRC-1 and CBP on breast cancer cell phenotype and gene expression, we created stable clones expressing these coactivators. Relative mRNA expression of SRC-1 and CBP in stable MCF7 and MDA-MB-468 clones is shown in Figure 3A and 3B. SRC-1 or CBP expression was increased by 2-6 fold when compared to neomycin resistant control clones. We also examined expression of SRC-1 and CBP proteins in these clones (Figure 3C). To determine the effects of coactivator overexpression on the chromatin organization of the proximal EGFR promoter, we performed chromatin immunoprecipitation using genomic DNA from stable clones. As shown in Figure 3D-G, PPAR and RXR ligands failed to recruit SRC-1 or CBP from the EGFR promoter in MCF7 and MDA-MB-468 cells overexpressing these coactivators. This was in marked contrast to the coactivator recruitment observed in parental cell lines and neomycin resistant control clones. PPAR and RXR ligands also failed to inhibit acetylation of histone H3 in the proximal EGFR promoter in SRC-1 or CBP overexpressing clones. Minimal nucleosomal DNA was amplified from these clones, indicating an open and transcriptionally permissive chromatin structure at the proximal EGFR promoter. These results indicated that SRC-1 and CBP acetylate histones in the proximal EGFR promoter, which correlated with resistance to PPAR and RXR ligands. To correlate these coactivator mediated changes with alterations in cell cycle regulatory genes, we examined protein expression by western blot. As shown in Figure 3H, MCF7 clones overexpressing SRC-1 and CBP showed no decreases in expression of EGFR, ERK1, c-myc, cdk1, and cdk4 in response to PPAR and RXR ligands as observed in parental cell lines and control clones. A slight reduction of cyclin A expression in response to retinoid and ciglitazone treatment was detected was observed in MCF7 clones. Overall similar results were observed in MDA-MB-468 clones. We also examined protein expression of the differentiation markers casein and WAP in SRC-1 and CBP overexpressing clones. SRC-1 and CBP overexpression dramatically inhibited expression of the differentiation marker casein by up to 16 fold in MCF7 clones (Figure 3I). Casein expression was not detected in MDA-MB-468 cells, nor was WAP protein expression observed in these clones by western blot. These results indicate that coactivator overexpression inhibits PPAR and RXR ligand mediated reductions in cell cycle regulatory gene expression.

**Figure 4:**
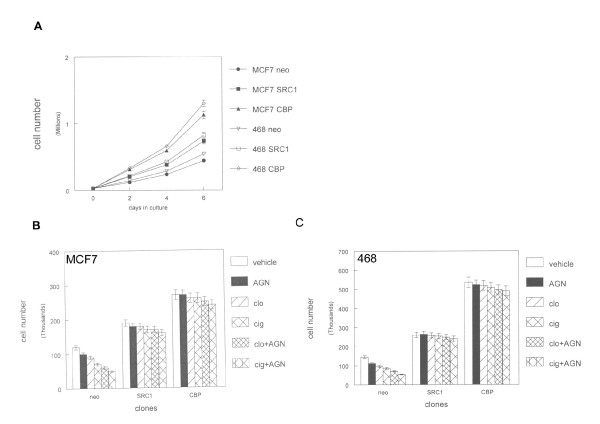
**Coactivator overexpression increases proliferation and resistance to PPAR ligands in human breast cancer cell lines**. (A) Triplicate cultures of stable CBP and SRC-1 expressing clones were grown in vitro for 6 days and compared to proliferation rates of neomycin resistant control cells (neo). Error bars indicate SEM of 3 independent experiments. (B, C) SRC-1, CBP, and neomycin resistant (neo) stable MCF7 or MDA-MB-468 clones were treated with 100 μM clofibrate (clo) or ciglitazone (cig) alone or in combination with 100 nM AGN194204 and grown in vitro for 6 days. Cells were counted with a hemacytometer. Error bars indicate SEM of 3 independent experiments.

We also determined the effects of coactivators on breast cancer cell proliferation. Both SRC-1 and CBP stable clones exhibited increased proliferation in vitro compared to MCF7 and MDA-MB-468 control clones (Figure 4A). SRC-1 overexpression increased cell numbers after 6 days culture by 60%. CBP stable clones exhibited 120% greater cell numbers after 6 days culture compared to control clones. PPAR and RXR ligands individually and in combination inhibited MCF7 and MDA-MB-468 control clone proliferation by 20-50% after 2 days treatment (Figure 4B, C). In contrast, PPAR and RXR ligands did not significantly inhibit cellular proliferation in SRC-1 and CBP stable clones. We concluded that coactivator overexpression increased breast cancer cell proliferation and blocked the inhibitory effects of PPAR and RXR ligands.

## Discussion

The results of our present study demonstrated that selective activation of PPARs and RXRs inhibits proliferation of human breast cancer cell lines in vitro. This inhibition of proliferation is reflected in decreased expression of cell cycle regulatory genes in treated breast cancer lines. These results extend the findings of our previous study using PPAR lipid ligands [22]. The large number of diverse PPAR ligands has produced pleiotropic effects in human breast cancer cell lines and animal tumor models. PPARγ ligands inhibited proliferation of human breast cancer cell lines in vitro [34], but individual lines may respond differently to the same drug [35]. PPARα activation by clofibrate has been shown to increase proliferation of breast cancer cells and of normal rat mammary gland in vivo [36,37]. However, both troglitazone (a PPARγ ligand) and clofibrate inhibited carcinogen induced mammary tumors in rats, in agreement with our results [38]. A previous study also showed that selective PPAR activation regulated histopathologic differentiation of mammary tumors [39]. While we did not observe changes in histopathologic pattern as the result of PPAR ligand exposure in the MMTV-neu mouse model, loss of SRC-1 produced increased expression of differentiation markers in these mammary tumors (see below).

Members of the EGFR gene family have been targets of molecular therapy for breast cancer. The results of our study indicated that EGFR is a target gene for PPAR and RXR antiproliferative therapy. Inhibition of the EGFR gene in human breast cancer cells is likely due to recruitment of CBP and SRC-1 from the EGFR promoter by ligand activated PPARs and RXRs. This recruitment correlates with decreased histone acetylation and nucleosome formation on the proximal EGFR promoter. In contrast, overexpression of SRC-1 or CBP induced resistance to the antiproliferative effects of PPAR and RXR ligands and blocks inhibition of EGFR expression by these compounds. Overexpression of the coactivator SRC-3 enhanced estrogen stimulated proliferation and blocked inhibition by anti-estrogens in human breast cancer cell lines [40]. SRC-1 or CBP overexpression increased proliferation of human breast cancer cell lines, which correlated with increased histone acetylation and loss of nucleosome formation on the EGFR promoter. Interestingly overexpression of either SRC-1 or CBP produced increased occupancy of the EGFR promoter by the other coactivator. In support of these findings, CBP and SRC-1 have been shown to synergistically interact to increase gene transcription [41]. In vivo, increased SRC-1 and EGFR expression was demonstrated in post-lactating rat mammary gland [42]. Conversely SRC-1 deficiency resulted in decreased hormone response in the null mutant mouse [15,16].

We also reported the effects of SRC-1 null mutation on mammary tumorigenesis in the MMTV-neu transgenic mouse. Loss of SRC-1 expression dramatically increased tumor latency in this model, which correlated with decreased proliferation index and upregulation of cell cycle inhibitors such as transforming growth factor β2 (5.2 fold), Cdkn1a (8.8 fold), and Cdkn2b (8.5 fold). Global gene expression profiling of these tumors revealed increased expression of differentiation markers, such as parotid secretory protein, salivary amylase 1, submaxillary gland androgen regulated protein, prolactin induced protein, cysteine rich secretory protein 1, whey acidic protein, and demilune cell and parotid protein. Upregulation of these genes indicate that SRC1-/- mammary tumors are more differentiated than their SRC1+/+ counterparts. Loss of SRC1 had differential effects on some classes of genes, such as upregulation of mucin 1 and casein alpha s2-like A (17.2 and 13.8 fold respectively) but downregulation of mucin 10 and casein alpha s2-like B (-3.9 and -5.9 fold). Interestingly a tissue specific knockout of PPARγ altered the lipid composition of mammary gland secretions with deleterious effects on neonatal mice [43]. Taken together, these results suggest that SRC-1 is a key regulator of gene products comprising mammary secretions. While expression of differentiation genes are not routinely determined in clinical cases of human breast cancer, Her2/neu and estrogen receptor levels are often used to determine if patients should be treated with targeted therapies against these gene products.

Expression of genes commonly altered in human breast cancer was also changed in SRC1-/- mouse mammary tumors. Bone morphogenetic protein 4 stimulates outgrowth of normal mammary buds [44], is upregulated in human breast cancer [45], and was increased by 6.1 fold in the SRC1-/- tumor model. However the role of bone morphogenetic protein 4 in human breast cancer is not clear, producing opposing effects on different human breast cancer cell lines. Expression of the Notch ligand Delta-like 1 is required for transformation of human mammary epithelial cells [46], and was 5 fold increased in the SRC1-/- mouse model. In this case upregulation of Delta-like 1 expression may help to compensate for loss of SRC1 in mammary tumorigenesis. Inhibition of Janus kinase 1 activation has been observed in human breast cancer [47], and its expression was downregulated by -3.5 fold in SRC1-/- mammary tumors. Members of the claudin family of tight junction proteins are epigenetically silenced in human breast cancer [48]; expression of claudin 10 was decreased by -81.2 fold in SRC1-/- mammary tumors. These results indicate that mammary tumors in the SRC1-/-;neu mouse recapitulate a number of gene expression changes in human breast cancer.

SRC-3 is another member of the SRC gene family, which is specifically amplified in breast cancer [49]. The SRC-3 null mutation inhibits mammary gland development, partly due to decreased estrogen levels in these mice [50]. Previous studies demonstrated that mice lacking SRC-3 were resistant to chemical carcinogen and ras oncoprotein induced mammary carcinogenesis [51], similar to the results of our present SRC-1 study. Since both SRC-1 and SRC-3 can interact with PPAR and RXR, the similar molecular mechanism by which ligand activated PPAR and RXR recruit SRC-1 and CBP from the EGFR promoter may also partially explain the promoting effect of SRC-3 overexpression in mammary tumorigenesis. On the other hand, the role of SRC-1 as a transcriptional coactivator of PPAR and RXR may also contribute to the PPAR/RXR mediated suppression of mammary tumorigenesis since SRC1-/- tumors were less sensitive to the inhibitory effects of PPARγ and RXR ligands. This expectation is consistent with a previous study showing that haploinsufficiency of AIB3 (NCOA6), a coactivator required for PPAR and RXR transcriptional activity, accelerated mammary tumor development in MMTV-polyoma middle T antigen mouse model and the AIB3+/- tumors also were less sensitive to the inhibitory effects of PPARγ and RXR ligands [52]. However, SRC-1 expression was not required for the peroxisome proliferation response to fibrates in the liver [53]. Expression of the PPARγ coactivator 1α (PGC1α) was downregulated in SRC1-/- tumors by -7.2 fold, which may contribute to the decreased sensitivity of these tumors to PPAR ligands.

In summary, histone acetyltransferases SRC-1 and CBP mediate resistance to nuclear receptor ligands and increased proliferation in human breast cancer cells. SRC-1 also plays a key role in initiation of mammary tumorigenesis in vivo, which is associated with changes in gene expression important to human breast cancer. Some of the limitations of the study include downregulation of PCG1α in SRC1-/-;neu mice which recruits other histone acetyltransferases such as CBP to PPARs. Additionally the effects of other mammary tumor promoting oncogenes such as Wnt1 and ras may alter the effects of SRC1 deletion. Future experiments will use these model to further dissect molecular signaling pathways relevant to human breast cancer.

## Conclusions

SRC-1 null mutation increased mammary tumor latency and decreased metastasis in neu transgenic mice and reduced the inhibitory effects of PPAR and RXR ligands. SRC1-/- mammary tumors were less proliferative and more differentiated with respect to gene expression than SRC1+/+ cancers. PPAR and RXR selective ligands were effective inhibitors of breast cancer cell proliferation in vitro. PPAR and RXR ligands inhibited multiple overlapping cell cycle regulatory proteins in human breast cancer cell lines. PPAR and RXR ligands inhibited interaction of coactivator proteins with key growth regulatory genes such as EGFR. Coactivators induced resistance to inhibition of EGFR promoter activity by nuclear receptor ligands. Coactivator overexpression increased cell proliferation and induced resistance to growth inhibition by PPAR and RXR ligands, which correlated with cell cycle regulatory gene expression. SRC-1 and CBP acetylated histones in the proximal EGFR promoter, which correlated with increased gene expression and resistance to the growth inhibitory effects of PPAR and RXR ligands.

## Abbreviations

PPAR: peroxisome proliferator activated receptors; RXR: retinoid X receptor, PBS: phosphate buffered saline; EGFR: epidermal growth factor receptor; CBP: CREB binding protein; SRC-1: steroid receptor coactivator 1.

## Competing interests

The authors declare that they have no competing interests.

## Authors' contributions

JSH performed experiments. DLC provided oversight of the research and manuscript writing. Both authors have read and approved the final manuscript.

## Pre-publication history

The pre-publication history for this paper can be accessed here:

http://www.biomedcentral.com/1471-2407/10/629/prepub
